# Impact of Sulfoxaflor on Brown Marmorated Stink Bug: Developmental and Reproductive Effects

**DOI:** 10.3390/insects16050465

**Published:** 2025-04-28

**Authors:** Ren Li, Zehua Wang, Fan Yang, Tao Su, Guanghang Qiao, Shanning Wang

**Affiliations:** 1 Institute of Plant Protection, Beijing Academy of Agriculture and Forestry Sciences, Beijing 100097, China; liren@baafs.net.cn (R.L.); wangzehua200707@163.com (Z.W.); evelynyangfan@163.com (F.Y.); s2678143384@163.com (T.S.); qghang98@126.com (G.Q.); 2 Key Laboratory of Environment Friendly Management on Fruit and Vegetable Pests in North China (Co-Constructed by the Ministry and Province), Ministry of Agriculture and Rural Affairs, Beijing 100097, China

**Keywords:** *Halyomorpha halys*, sulfoxaflor, sublethal effects

## Abstract

The brown marmorated stink bug, *Halyomorpha halys*, has become a severe threat to agriculture worldwide with continuous invasion and spread. Sulfoxaflor is the pioneering sulfoximine insecticide and has been widely employed for managing sap-feeding insect pests across diverse crops. However, the sublethal effects of sulfoxaflor on the growth and reproduction of *H. halys* remain unexplored. This study employed age-stage life tables and physiological parameters to evaluate the sublethal effects of sulfoxaflor on *H. halys*. The bioassay results indicated that the LC_20_ and LC_50_ values of sulfoxaflor against second-instar nymphs of *H. halys* were 7.75 and 20.97 mg/L, respectively. Treatment with the LC_20_ concentration of sulfoxaflor reduced the lifespan of female adults by 11.74 days. Importantly, the oviposition period of females was shortened by 9.47 days, and the fecundity was decreased by 1.4-fold. Additionally, the activities of cytochrome P450 (P450s) and glutathione *S*-transferase (GSTs) enzymes in *H. halys* were enhanced 1.40 and 1.54 times after treatment with sublethal concentrations of sulfoxaflor. These findings demonstrate that sublethal concentrations of sulfoxaflor significantly inhibit the population development of *H. halys*. Moreover, the detoxification enzymes P450s and GSTs may be associated with the potential resistance to sulfoxaflor in *H. halys*. This information provides valuable insights into the application of sulfoxaflor in the management of this pest.

## Simple Summary

The brown marmorated stink bug is an invasive pest that causes significant damage to agriculture worldwide. It feeds on a wide variety of crops, leading to substantial economic losses. To manage this pest, farmers often rely on chemical insecticides. However, the effectiveness of these chemicals can be reduced when pests are exposed to sublethal doses, which may not kill them outright but can still affect their growth and reproduction. This study investigates the effects of a specific insecticide of sulfoxaflor on the brown marmorated stink bug. We found that even at low concentrations, sulfoxaflor can reduce the lifespan of females, shorten the period during which females lay eggs, and decrease the number of eggs laid. Additionally, the insecticide can increase the activity of certain enzymes in the bug that help it detoxify harmful substances, potentially leading to resistance. These findings suggest that sulfoxaflor can be an effective tool for controlling the brown marmorated stink bug, but its use should be carefully managed to prevent the development of resistance. This research provides valuable insights for developing sustainable pest management strategies, helping protect crops and reduce the economic impact of this invasive pest.

## 1. Introduction

The brown marmorated stink bug, *Halyomorpha halys* (Stål) (Hemiptera: Pentatomidae), which is native to East Asia, has invaded North America and Europe and become a globally severe threat to agriculture [1]. The host plants of the brown marmorated stink bug exceeded 300 species, and it prefers economically important tree fruits, vegetables, field crops, and ornamentals, causing severe economic losses [2,3,4,5]. *H. halys* causes damage to fruits by nymphs and adults aggregating and feeding on the juice, resulting in white spongy areas on the fruit peel and internal damage; it can also lead to the abortion of flower buds and immature fruits, thereby reducing fruit set and overall yield [5]. In China, the brown marmorated stink bug primarily infests apple, pear, kiwifruit, and peach trees in orchards. The status of *H. halys* has shifted from an occasional outbreak pest to a serious agricultural pest due to its adoption and subsequent spread. Data indicate that the damage inflicted by *H. halys* in orchards of Northern China can surpass 30%, posing a substantial challenge to the advancement of ecological orchard practices [6].

The control of *H. halys* has predominantly relied on chemical control. The efficacy of insecticides is typically gauged by the mortality rate of pest populations within a given time frame [7,8]. However, insecticides can also influence population dynamics through non-lethal means. The sublethal effects of pesticides can provide a more precise estimation of overall insecticide efficacy. Many insecticides tested on *H. halys* do not result in a high rate of direct adult mortality, even at the maximum recommended rates [9]. The high mobility of adult *H. halys* allows for rapid dispersal from treated areas, potentially reducing their exposure to lethal doses [10,11]. Nevertheless, these insecticides may still have significant sublethal effects, altering the physiology, behavior, and biochemistry of pests, including adverse changes in survival rate, development duration, fecundity, and longevity [12,13]. Therefore, assessing the sublethal effects of insecticides could provide a more accurate measure of their efficacy against *H. halys*.

Sulfoxaflor, a sulfoximine insecticide, acts on the nicotinic acetylcholine receptors (nAChRs) and exhibits high toxicity against a broad range of piercing–sucking pests, including aphids, whiteflies, and hoppers [14]. Moreover, it does not exhibit cross-resistance with neonicotinoid insecticides such as imidacloprid [14,15,16]. The sublethal effects of sulfoxaflor have been assessed in *Nilaparvata lugens*, *Panonychus citri*, *Sogatella furcifera*, and *Aphis gossypii*, and these results show that the sublethal doses reduce the reproductive capacity of pest populations [17,18,19].

However, sulfoxaflor is not registered for the control of *H. halys* in China, and the sublethal effects of sulfoxaflor against *H. halys* have not been systematically evaluated. This restricts the application of sulfoxaflor in the management of *H. halys*. In this study, we determined the toxicity of sulfoxaflor to *H. halys* and evaluated the effects of sublethal concentrations of sulfoxaflor on the life table parameters of *H. halys* using life table methods. Furthermore, we investigated the impact of sulfoxaflor on the activities of three major detoxification enzymes. Our findings provide a theoretical foundation for the effective utilization of sulfoxaflor in the management of *H. halys* within agricultural contexts.

## 2. Materials and Methods

### 2.1. Test Insects

In October 2019, *Halyomorpha halys* were collected from ash trees in the suburbs of Beijing, China, in which sulfoxaflor had never been applied. The collected insects were maintained in laboratory cages (30 × 30 × 30 cm) within an incubator set at 25 ± 1 °C, with 60% relative humidity and a 16 h:8 h light–dark photoperiod. The insects were fed with organic snap bean pods and corn, which were replaced three times per week, and provided with deionized water. Healthy 2nd-instar nymphs were selected for the laboratory experiments.

### 2.2. Insecticide Formulation

The commercial formulation of sulfoxaflor (22% WP) was obtained directly from the manufacturer (Dow AgroSciences, Johnston, IA, USA). This formulation was subsequently serially diluted into five specific concentrations using deionized water containing 0.1% Triton X-100 (Sigma-Aldrich, Saint Louis, MO, USA). The concentrations prepared for the toxicity bioassays were 100, 50, 25, 12.5, and 6.25 mg/L.

### 2.3. Toxicity Test

The toxicity was assessed using the snap bean pod dip method with slight modifications [20]. Fresh and untreated snap bean pods were individually emerged in the solutions for 10 min and then left to air-dry. Once dry, the pods were placed inside a plastic box (6.0 cm in diameter). Because the first-instar nymphs of *H. halys* aggregate on the eggshell and do not feed on fruits, and only begin to feed on fruits from the second-instar stage, the second-instar nymphs were selected for this study. After a 12 h starvation period, twenty 2nd-instar nymphs were introduced into the box, which was subsequently sealed with a lid. All treated samples were maintained in an incubator calibrated to 25 ± 1 °C, with a relative humidity maintained at 60–70% and a 16 h:8 h light–dark photoperiod. Mortality was assessed after 48 h by counting the number of deceased nymphs. Nymphs that were unable to move were considered dead. Each treatment was replicated four times, with deionized water containing 0.1% Triton X-100 serving as the control. Data were analyzed using probit analysis using PoloPlus 1.0 software (LeOra Software Inc., Berkeley, CA, USA).

### 2.4. Sublethal Effects of Sulfoxaflor on H. halys

A life table was constructed to evaluate the effects of the sublethal concentration (LC_20_) of sulfoxaflor on the growth, development, and reproduction of *H. halys*. Egg masses of *H. halys* were reared until the nymphs reached the 2nd-instar stage. Snap bean pods were immersed in a sulfoxaflor solution at the LC_20_ concentration (7.75 mg/L) for 10 min and used as food sources. Second-instar nymphs, which were starved for 12 h, were individually placed into plastic boxes (6.0 cm in diameter) and provided with the treated snap bean pods. After 48 h, surviving nymphs were fed fresh, untreated snap bean pods, which were replaced daily until the nymph reached adulthood.

The calculation methods for the life table parameters were based on the approach described by Liu et al. (2006) [21]. For each nymph, mortality and developmental duration were monitored at 24 h intervals. The survival rates from the 2nd to the 3rd instar (Sr1) and from the 3rd to the 5th instar (Sr2) were assessed as the nymphs progressed. Upon reaching adulthood, the emergence rate (Er) and female ratio (Fr) were documented. Subsequently, females and males were paired for reproduction. Following oviposition, egg masses were transferred daily to separate plastic boxes, and fecundity (Fd) was measured as the average number of eggs laid by mated females. Females that failed to produce offspring were considered unsuccessful in copulation, and the copulation rate (Cr) was calculated accordingly. The hatchability of egg masses (Ha) was also recorded. Adult longevity was monitored daily until death. This study included three replicates, with 60 nymphs evaluated per replicate. Deionized water containing 0.1% Triton X-100 was used as the control.

The population trend index (I) and relative fitness were derived using the following formula: Nt = N × Sr_1_ × Sr_2_ × Er × Fr × Cr × Fd × Ha, I = N_t_/N_0_. Here, N_0_ represents the initial population count and Nt denotes the population count of the subsequent generation. To identify statistical differences between treatments, data were analyzed using the independent-samples *t*-test with a significance threshold of *p* = 0.05. All statistical analyses were performed using SPSS 16.0 (IBM, Chicago, IL, USA).

### 2.5. Enzyme Assays

Second-instar nymphs exposed to the LC_20_ concentration of sulfoxaflor were evaluated for the activity of three detoxifying metabolic enzymes. Carboxylesterase (CarEs) activity was measured using the Carboxylesterase test kit (Nanjing Jiancheng Bioengineering Institute, Nanjing, China) following the manufacturer’s instructions. The assay is based on the principle that CarEs catalyzes 1-naphthyl acetate to produce α-naphthol, which then undergoes a colorimetric assay via the diazotization reaction with fast B salt. The presence of CarEs is indicated by a solid blue color, with light absorption at 450 nm reflecting the formation rate of naphthol ester. This allows for the calculation of CarEs activity at 37 °C. To prepare the samples, five 2nd-instar nymphs were quickly transferred into a centrifuge tube and flash-frozen in liquid nitrogen. The samples were then homogenized in a 600 µL phosphate buffer (0.04 mol/L of phosphate buffer, pH 7.0) ice-bath, followed by centrifugation at 4 °C and 12,000 g for 30 min. The supernatant was collected into a clean Eppendorf tube as the crude enzyme extract. According to the manual’s protocol, the absorbance was measured at 405 nm at 10 and 190 s using a UV 2000 spectrophotometer (Unico, Shanghai, China). An increase of one unit in the catalytic absorbance value per milligram of tissue protein per minute at 37 degrees is defined as 1U.

Glutathione *S*-transferase (GSTs) activity was assessed using the GST Assay kit (Nanjing Jiancheng Bioengineering Institute, Nanjing, China). This kit operates on the principle that GST catalyzes the conjugation of L-glutathione to 1-chlom-2,4-dinitrobenzene (CDNB) via the thiol group of glutathione, producing the GS-DNB conjugate, which exhibits absorbance at 340 nm. The absorption rate increase corresponds directly to the GST activity present in the sample.

Cytochrome P450 (P450s) activity was measured using the CYP450 EKISA kit (Realbio-Tech, Shanghai, China), which is based on a color change from blue to yellow upon the addition of a Stop Solution. The intensity of the yellow color, measurable at 450 nm, reflects the P450 activity in the sample.

For each enzyme assay, three biological replicates were prepared, and each sample was technically replicated three times. The total protein content in the enzyme solutions was quantified using the Bradford method, with bovine albumin serving as a standard [22].

### 2.6. Data Analysis

The significance analysis of differences in adult female lifespan and egg production was conducted using Student’s *t*-test (*p* < 0.05). The activities of CarEs, GSTs, and P450s were statistically analyzed using the unpaired Student’s *t*-test, with a significance threshold set at *p* < 0.05.

## 3. Results

### 3.1. Toxicity of Sulfoxaflor Against H. halys

The bioassay revealed that the LC_20_ and LC_50_ values of sulfoxaflor against 2nd-instar nymphs of *H. halys* were 7.75 and 20.97 mg/L, respectively (Table 1). The LC_20_ of sulfoxaflor was used for the subsequent sublethal exposure experiments.

### 3.2. Sublethal Effects of Sulfoxaflor on Nymph Development of H. halys

The effects of the LC_20_ concentration of sulfoxaflor on the development time of *H. halys* nymphs are summarized in Table 2. Sulfoxaflor significantly prolonged the developmental duration of 2nd- and 3rd-instar nymphs at the LC_20_ treatment (2nd instar: *F* = 3.92, *df* = 245, *p* < 0.001; 3rd instar: *F* = 3.48, *df* = 245, *p* = 0.011). However, no statistically significant differences were observed in developmental periods of the 4th and 5th instars compared to the control. Furthermore, there was a significant difference in the total developmental duration of nymphal stages between the treatment and control groups (*F* = 5.57; *df* = 245; *p* < 0.001), with the LC_20_ of sulfoxaflor causing a delay of 2.29 days in the total nymphal stages.

### 3.3. Effects of Sulfoxaflor on Longevity and Reproduction of Adults

**Figure 1:**
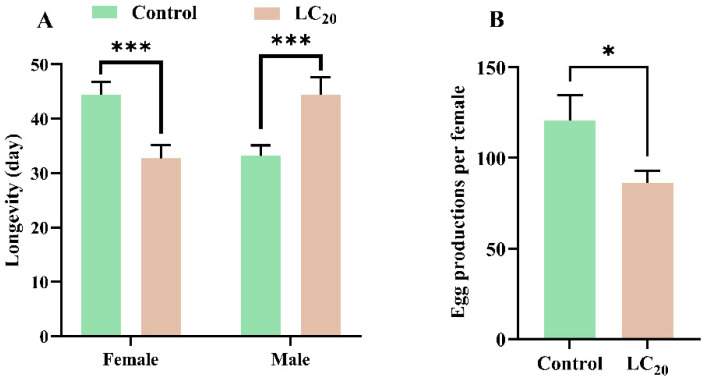
Effect of sulfoxaflor at LC_20_ to *Halyomorpha halys* adult longevity (**A**) and fecundity (**B**). Data are represented by mean ± SE, and “***” and “*” indicate *p*-values lower than 0.001 and 0.05, respectively.

The impact of sublethal concentrations of sulfoxaflor on the longevity of the *H. halys* was analyzed across treatments (Figure 1). Exposure to LC_20_ concentrations of sulfoxaflor resulted in a decrease in female longevity from 44.45 days to 32.71 days (*F* = 0.032; *df* = 121; *p* < 0.001). Conversely, male longevity increased from 33.14 d to 44.40 d (*F* = 11.98; *df* = 64.85; *p* = 0.004). The fecundity of females in the LC_20_ treatment was significantly reduced by 1.4 times compared with the control (Figure 1).

The preoviposition period of *H. halys* adult females in the control group was longer than the sublethal concentration of sulfoxaflor treatment (*p* < 0.01), and the oviposition period in the LC_20_ treatment group was significantly reduced by 9.47 days compared with the control (Table 3).

### 3.4. Effects of Sulfoxaflor on Life Table Parameter of H. halys

The sublethal concentration of sulfoxaflor showed negative effects on *H. halys* life table parameters (Table 4). The survival rates from the 2nd to 3rd and 3rd to 5th instar ages in the treatment groups were 0.76 and 0.90, respectively, compared to 0.93 in the control group. Notably, the treatment of sublethal sulfoxaflor concentrations resulted in a reduced nymph survival rate, adult emergence rate, copulation rates, and hatchability compared to the control. Consequently, the overall population fitness declined to 0.56 relative to the control treatment.

### 3.5. Effects of Sulfoxaflor on Enzymatic Activity of H. halys

**Figure 2:**
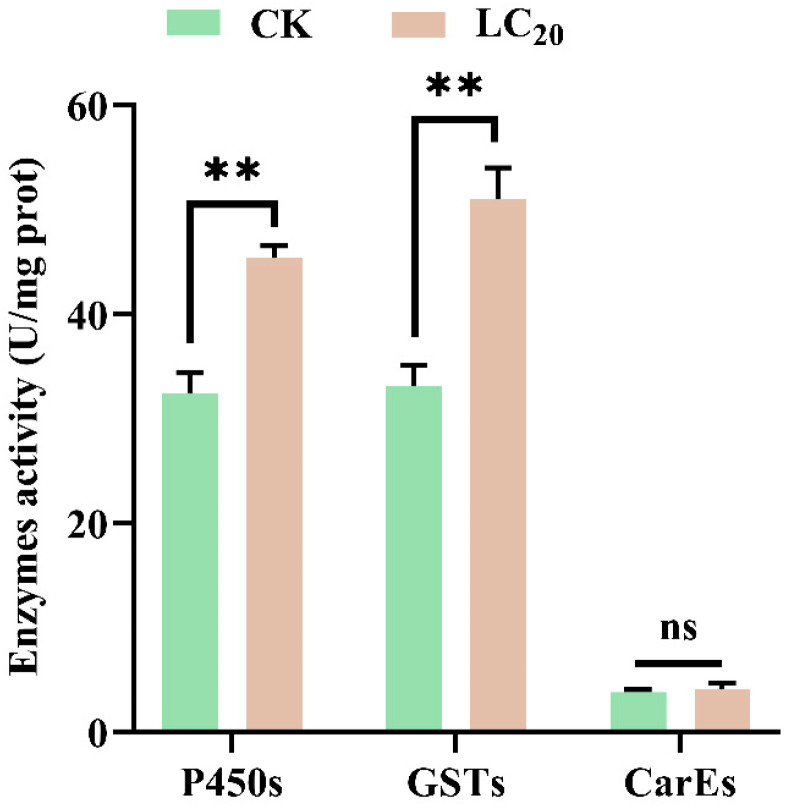
The detoxification enzyme activities of *Halyomorpha halys* after treatment with LC_20_ concentrations of sulfoxaflor. Data are presented as the mean ± SE. “**” and “ns” indicate that the *p*-value was lower than 0.01 and greater than 0.05, respectively.

The impact of sublethal concentrations of sulfoxaflor on the detoxification enzyme activities of CarEs, GSTs, and P450 in 2nd-instar nymphs of *H. halys* is illustrated in Figure 2. The activities of GSTs and P450s in nymphs exposed to the LC_20_ concentration of sulfoxaflor were significantly increased by 1.40 and 1.54 times compared with the control group, but CarEs activity showed no significant difference (*p* = 0.591).

## 4. Discussion

With continuous invasion and expansion, *H. halys* has posed a threat to the safe production of global agriculture [1]. Chemical control is the primary method for the management of this pest, and pyrethroids and neonicotinoids have been widely used for its control [8,23,24]. Sulfoxaflor is a competitive modulator of nAChRs and has been classed into a distinct 4C group by the Insecticide Resistance Action Committee (IRAC) [25] Sulfoxaflor has exhibited a high efficiency for sap-sucking pests, such as *A. gossypii*, *N. lugens*, and *Bemisia tabaci* [14,16]. Our results showed that sulfoxaflor has toxicity against *H. halys*, with an LC_50_ value of 20.97 mg/L. The toxicity of sulfoxaflor to *H. halys* was similar to the toxicities of imidacloprid, dinotefuran, thiamethoxam, abamectin, λ–cyhalothrin against 2nd-instar nymphs, with the LC_50_ ranging from 3.16 to 42.11 mg/L, as determined in our previous study [20].

However, insects may encounter sublethal concentrations of insecticides due to the degradation of these chemicals by biotic and abiotic factors. The exposure of insects to sublethal doses of insecticides usually resulted in the generation of fitness costs. Some studies have revealed that exposure to sublethal doses of sulfoxaflor significantly reduces key population parameters in *Apolygus lucorum*, *N. lugens*, and *S. furcifera*, including the intrinsic rate, finite rate, mean generation time, net reproductive rate, and gross reproduction rate, ultimately affecting population reproduction [18,26]. In this study, the results indicated that the female longevity and oviposition period were reduced by 11.74 and 9.47 days, and the fecundity significantly declined by 1.4-fold in the treatment of sublethal doses of sulfoxaflor. Additionally, population analysis showed a significant decline in nymph survival rate, adult emergence rate, and mating rate when *H. halys* nymphs were exposed to an LC_20_ concentration of sulfoxaflor. Consequently, the population fitness was reduced to 56% of that of the control group. This suggests that the exposure to sublethal doses of sulfoxaflor in *H. halys* suppresses population reproduction.

Current research has revealed from the molecular mechanism perspective that sublethal doses of sulfoxaflor significantly affect the ovarian development of female insects, thereby impacting their reproduction. Vitellogenin (Vg) is a critical phospholipid glycoprotein involved in vitellogenesis across nearly all oviparous animals. By binding to the vitellogenin receptor (VgR), Vg promotes the formation of yolk, which is crucial for the normal development of ovaries in adult females [27]. In the study of *P. citri*, exposure to the LC_30_ concentration of sulfoxaflor was found to significantly increase the expression levels of Vg and VgR genes [17]. Meanwhile, it was determined that exposure to the LC_15_ concentration of sulfoxaflor had no significant effect on the expression level of Vg gene in the F0 generation in *A. lucorum*, but the expression level of Vg gene in the F1 generation was significantly reduced by 43.8% [26]. Similar studies have found that in *Coccinella septempunctata*, treatment with the sublethal application rates of sulfoxaflor (LR_10_ and LR_30_) significantly reduced the expression level of Vg gene in F0 generation by 63.59- and 65.38-fold, and the protein content of Vg declined by 1.24 and 1.35 times, respectively [28]. These findings highlight that sublethal doses of sulfoxaflor can impact the reproductive system of female insects, leading to changes in fecundity. Similar effects may also occur in *H. halys*, but further validation is needed.

Exposure to sublethal doses of insecticides typically affects the detoxification metabolism processes in insects. The key enzymes involved in the metabolism of xenobiotics are primarily P450s, GSTs, and CarEs [29,30,31]. Our study demonstrates that exposure to sublethal concentrations of sulfoxaflor significantly enhances the activities of P450s and GSTs by 1.40- and 1.54-fold in *H. halys*, whereas the activities of CarEs remain unchanged compared to the control. Previous studies have also determined that the sublethal concentration of sulfoxaflor induced an increase in activities of in P450 and GST in *P. citri* and *S. furcifera* [17,32]. Importantly, some research has demonstrated that the elevation in activities of P450s and GSTs was involved in the resistance to sulfoxaflor, and the P450 genes of *CYP6ER1*, *CYP6CY13*, *CYP6CY19*, and *CYP380C4* played a critical role in sulfoxaflor resistance in *A. gossypii*, *N. lugens*, and *Myzus persicae* [32,33,34,35]. This suggests that the enhancement in these detoxification enzymes may be associated with the potential development of resistance to sulfoxaflor in *H. halys*.

## 5. Conclusions

In conclusion, our results confirm that the exposure of *H. halys* to sublethal doses of sulfoxaflor suppresses population reproduction and elicits a positive response in pest control. However, it should be noted that continuous use may lead to the development of detoxification metabolic resistance. These findings provide a theoretical basis for the rational application of sulfoxaflor in the management of *H. halys*.

## Figures and Tables

**Table 1:** The toxicity of sulfoxaflor to the 2nd-instar nymphs of *Halyomorpha halys*.

Number	Slope ± SE ^a^	χ^2^ (*df*) ^b^	LC_20_ (95% CI ^c^) (mg/L)	LC_50_ (95% CI ^c^) (mg/L)
270	1.95 ± 0.33	4.32 (3)	7.75 (0.95–14.520)	20.97 (9.75–45.16)

^a^ SE, standard error; ^b^ Chi-square value (χ^2^) and degrees of freedom (*df*); ^c^ CI, confidence interval.

**Table 2:** Effects of the LC_20_ concentrations of sulfoxaflor on the developmental duration of nymphs of *Halyomorpha halys*.

Life Stage	Sulfoxaflor				Control				*p*-Value
	Number	Developmental Duration (Day)	Longest Period (Day)	Shortest Period (Day)	Number	Developmental Duration (Day)	Longest Period (Day)	Shortest Period (Day)	
2nd instar	125	10.82 ± 0.11	13	9	125	8.68 ± 0.12	12	6	<0.0001
3rd instar	125	7.26 ± 0.11	12	6	124	6.85 ± 0.11	11	4	0.011
4th instar	124	6.86 ± 0.22	13	4	124	7.19 ± 0.14	14	4	0.216
5th instar	120	10.24 ± 0.24	13	7	114	10.45 ± 0.17	14	6	0.495
Total nymph stage (except 1st instar)	120	34.91 ± 0.36	54	29	114	32.97 ± 0.30	27	47	<0.0001

**Table 3:** Effects of sulfoxaflor at LC_20_ to *Halyomorpha halys* on preoviposition period and oviposition period.

Developmental Period	LC_20_	CK
Preoviposition period (days)	14.98 ± 0.67 ^b^	19.51 ± 0.78 ^a^
Oviposition period (days)	19.53 ± 2.83 ^b^	29.00 ± 2.40 ^a^

Data are represented by mean ± SE, and different letters indicate *p*-values lower than 0.05 according to *t*-test analysis.

**Table 4:** Life table parameters of *Halyomorpha halys* after treatment with the LC_20_ concentration of sulfoxaflor.

Parameter	LC_20_	Control
N_0_	175	134
Sr1 (survival rate from 2nd to 3rd instar)	0.76	0.93
Sr2 (survival rate from 3rd to 5th instar)	0.90	0.93
Er (emergence rate)	0.87	0.96
Fr (female ratio)	0.61	0.55
Cr (Copulation rate, %)	0.79	0.93
Fd (Fecundity, eggs per female)	90.61	87.82
Ha (Hatchability, %)	0.75	0.95
N (predicted number of offspring)	3453.52	4711.19
I (population trend index)	19.73	35.19
Relative fitness	0.56	1

## Data Availability

The original contributions presented in this study are included in the article. Further inquiries can be directed to the corresponding author.
